# Long Atrial Fibrillation Duration and Early Recurrence Are Reliable Predictors of Late Recurrence After Radiofrequency Catheter Ablation

**DOI:** 10.3389/fcvm.2022.864417

**Published:** 2022-03-25

**Authors:** Zhitong Li, Shihao Wang, Tesfaldet H. Hidru, Yuanjun Sun, Lianjun Gao, Xiaolei Yang, Yunlong Xia

**Affiliations:** Department of Cardiology, First Affiliated Hospital of Dalian Medical University, Dalian, China

**Keywords:** atrial fibrillation, catheter ablation, recurrence, early recurrence, AF duration

## Abstract

**Background:**

Recurrence after atrial fibrillation (AF) ablation is still common.

**Objective:**

This study aimed to evaluate the predictive abilities of AF duration and early recurrence (ER) to discriminate high-risk patients for recurrence.

**Methods:**

We enrolled 1,763 consecutive patients with AF who were scheduled to receive the index radiofrequency catheter ablation (RFCA) from January 2016 to August 2021 in Dalian, China. Long AF duration (LAFD) was considered if the course of AF lasted for ≥ 12 months. ER was defined as any atrial tachycardia (AT) or AF event longer than 30 s occurring within a 3-month post-RFCA.

**Results:**

Late recurrence occurred in 643 (36.5%) of the 1,763 patients at a median of 35 months after RFCA. Multivariate analysis identified LAFD (hazard ratio (HR): 1.80, 95% confidence interval (CI): 1.38–2.35, *p* < 0.001) and ER (HR: 2.34, 95% CI: 1.82–3.01, *p* < 0.001) as strong independent predictors of late recurrence in non-paroxysmal AF. Similarly, LAFD (HR: 1.48, 95% CI: 1.20–1.84, *p* < 0.001) and ER (HR: 3.40, 95% CI: 2.68–4.30, *p* < 0.001) were significantly associated with late recurrence in paroxysmal AF. Receiver operating curve analyses revealed that the CAAP-AF (CAD, Atrial diameter, Age, Persistent or longstanding AF, Antiarrhythmic drugs failed, Female) had the highest predict power [area under ROC curve (AUC) 0.586]. The addition of ER and LAFD to the CAAP-AF score significantly improved risk discrimination for late recurrence after AF ablation from 0.586 to 0.686.

**Conclusion:**

Long AF duration and ER were independently associated with late recurrence. The prediction performance of the CAAP-AF model for recurrence was improved by the addition of LAFD and ER.

## Introduction

Atrial fibrillation (AF) is one of the most common arrhythmias, which affects 1 in 200 patients worldwide (1) and is associated with poor outcomes. Radiofrequency catheter ablation (RFCA) is a common and effective therapy for drug-refractory symptomatic patients with AF (2). Despite an obvious advantage in procedures and technology, the recurrence rate of AF after the ablation remained significant and ranged from 30 to 60% (3). Various risk factors for AF recurrence have now been identified, and several scoring systems based on antecedent cardiovascular events, comorbidities, and biomarkers for AF recurrence have been established (2). However, the discriminatory ability of these models is highly variable, and there are no widely used models for quantitative prediction of AF recurrence in patients who underwent RFCA (4).

Long AF duration (LAFD) before the ablation procedure, which is often considered as a surrogate marker of heavier AF burden and more advanced atrial remodeling, indicates a poor prognosis (5) and is independently linked to late recurrence (6, 7). Early recurrence (ER) is defined as any atrial tachycardia (AT) or AF event longer than 30 s occurring within a 3-month post-RFCA (which is known as the blanking period). ER is not considered a real recurrence in most studies (8, 9) because it is often considered as transient inflammation related to ablation, not affecting long-term results. However, ER is common after RFCA and has been associated with an increased risk of late AF recurrence (10–12). Although, a few prediction models have included LAFD and ER as the risk stratification factors of late recurrence.

In this study, we aimed (i) to assess the association between LAFD and ER observations and late recurrence and (ii) to verify whether the addition of LAFD and ER can improve the prediction efficiency of the previously recommended models in the guideline.

## Materials and Methods

### Study Participants

**FIGURE 1:**
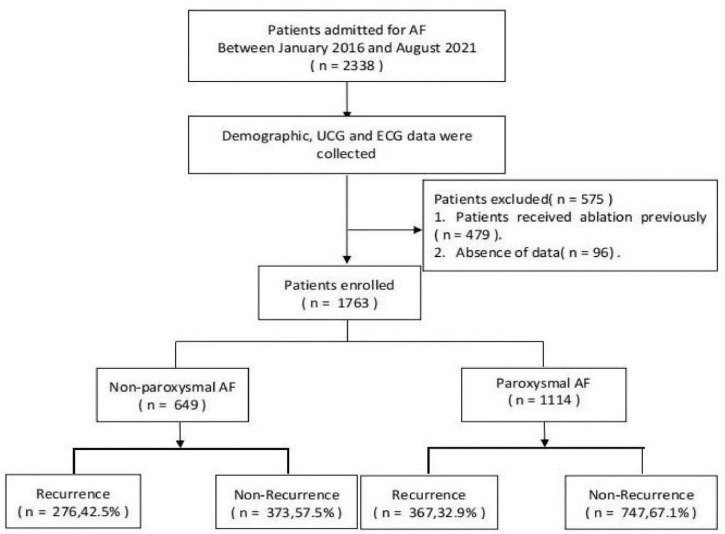
The overview of the selection of study participants.

This retrospective study enrolled 1,763 consecutive patients with AF who were scheduled to receive the index RFCA from January 2016 to August 2021 in the First Affiliated Hospital of Dalian Medical University (FAHDMU). Patients who had previously received ablation were excluded. Likewise, patients with missing/incomplete data were excluded. The flow chart of subject inclusion is summarized in Figure 1. The study was approved by the Institutional Review Board of the First Affiliated Hospital of the Dalian Medical University, and the requirement for informed consent was waived. The research was conducted according to the Helsinki declaration guidelines, and all procedures were carried out in accordance with the approved guidelines.

### Definition of the Explanatory Variables

Demographic, medical history, and laboratory data were obtained from the electronic medical record of FAHDMU. Diabetes mellitus (DM) was defined as a fasting glucose level ≥ 126 mg/dl (or random blood glucose level ≥ 200 mg/dl), a history of DM diagnosis by a physician or the use of diabetes medications (13). Congestive heart failure (CHF) was defined as a previous diagnosis of heart failure combined with the treatment with diuretics (14). Coronary artery disease (CAD) was defined if patients have at least one of the following characteristics: a previous diagnosis of CAD or the presence of ≥ 50% luminal stenosis in at least one major coronary artery in coronary angiography (15). Hypertension (HTN) was defined as systolic blood pressure ≥ 140 mmHg or diastolic blood pressure ≥ 90 mmHg at two or more visits or a past medical history of HTN or the use of antihypertensive medications (16). Dyslipidemia was defined if patients have one or more of the following conditions: TC ≥ 6.22 mmol/L, LDL-C ≥ 4.14 mmol/L, HDL-C ≤ 1.04 mmol/L, TG ≥ 2.26 mmol/L, or the use of anti-dyslipidemia medication (17). The definition and classification of AF were according to the guideline (18). Non-paroxysmal AF was composed of persistent, long-standing persistent, and permanent AF. The duration of AF was measured based on the length of time from the first diagnosis of AF based on electrocardiogram (ECG) to RFCA.

### Ablation Procedure

The preprocedural anticoagulant protocol and ablation procedure have been previously described (19). Three-dimensional LA and pulmonary vein (PV) anatomy were reconstructed by electroanatomic mapping systems (CARTO, Biosense Webster Inc., United States). Briefly, PV isolation was performed by ablation catheter (SmartTouch/SmartTouch Surround Flow, Biosense Webster, Inc., United States) using contiguous circumferential lesions guided by multispline catheter (PentaRay, Biosense Webster Inc., Diamond Bar, United States) or a circumferential mapping catheter (LASSO, Biosense Webster, Inc., United States). After initial circumferential pulmonary vein isolation (CPVI), if AF continued, linear ablation (LINE; left atrial roof, mitral isthmus, or tricuspid isthmus) was performed to achieve a bidirectional block or restore sinus rhythm. Then, complex fractionated atrial electrograms (CFAEs) were identified and ablated in the atrium and coronary sinus. The endpoint of ablation included the conversion of AF to sinus rhythm and the establishment of a bidirectional conduction block, which was demonstrated by pacing maneuvers, and voltage reduction or disappearance of CFAE. If AF was organized to AT, entrainment or activation mapping was performed to target and ablate the critical isthmus for macro-reentrant AT or the earliest activation of focal AT. If AF or AT continued despite the abovementioned ablation, external electrical or/and drug cardioversion was finally performed. The electrical power was set as bidirectional 200J, and the pharmacological cardioversion was carried out by ibutilide (1 mg) or amiodarone. If the triggered firing was noted in the superior vena cava (SVC), then the segmental isolation of the SVC was performed. According to the patient’s electronic medical record, the procedural strategies were categorized into CPVI alone, CPVI plus LINE, CPVI plus the ablation of CFAEs, and CPVI plus LINE plus CFAE (9).

### Post-ablation Management and Follow-Up

Oral anticoagulation therapy was continued for at least 3 months in all patients. Patients were recommended to visit the outpatient clinic regularly at 1, 3, 6, and 12 months and then annually or whenever symptoms occurred after the RFCA. All patients underwent ECG and 24-h Holter recording during each visit. Outcome data were obtained from outpatient visits and the hospital database system. AF recurrence was defined as any episode of AF/AT lasting for more than 30s. Recurrence within a 3-month blanking period was considered as ER, and an AF recurrence after 3-month post-RFCA was considered as late recurrence.

### Statistical Analysis

Statistical analyses were performed using R software (version 3.3.0). For continuous data, normality was evaluated with the Shapiro–Wilk test. Because all the continuous variables were not normally distributed (*p* < 0.05), these variables are presented as medians (interquartile range) and compared using the Mann–Whitney test. The categorical data were presented as frequencies (percentages) and analyzed by the χ^2^-test or Fisher exact test. All analyses were stratified by AF type due to different atrial substrates between the paroxysmal and non-paroxysmal AF. We first defined the cut point of AF duration by restricted cubic splines (RCSs). The number of knots was determined by the minimum value for the Akaike information criterion (AIC). Finally, we chose 4 knots located at the 5th, 35th, 65th, and 95th percentiles of AF duration. A hazard ratio (HR) = 1 indicates a reference value (cut point), HR greater than 1 indicating a higher risk of recurrence, and HR less than 1 indicating a lower risk of recurrence.

We further calculated HR and corresponding 95% confidence intervals (CIs) for the AF recurrence associated with LAFD and ER. Considering the potential confounding factors, we adjusted for the risk factors for AF recurrence identified from the guideline, including age, smoking, sex gender, HTN, estimating glomerular filtration rate (eGFR), CAD, DM, ER, body mass index (BMI), left atrial dimension (LAD), and left ventricular ejection fraction (LVEF) (2). We used three different models: Model 1, crude model; Model 2 was adjusted for age and sex; and Model 3 was adjusted for all the aforementioned variables. Kaplan–Meier curves were used to estimate the freedom from late recurrence with a log-rank test used to discriminate between Kaplan–Meier curves.

To evaluate the discriminatory power of AF recurrence at 5 years among different models in guideline (2), we invited time-dependent Receiver operating characteristics (ROC) curves, Harrell’s concordance statistics (*C*-statistics), net reclassification index (NRI), and integrated discrimination improvement (IDI). To evaluate the performance of the final combined model (CAAP-AF + LAFD + ER), we used the calibration plot with 1,000 bootstrap samples to decrease the overfit bias. In addition, a decision curve analysis (DCA) was performed to evaluate the clinical benefit of our final model. *P* < 0.05 was considered statistically significant.

## Results

### Baseline Characteristics

A total of 1,763 patients were enrolled in the study with a median age of 63 years. During a median follow-up of 35 months, 643 patients (36.5%) experienced AF recurrence. The baseline characteristics of the included patients are shown in Table 1. The rate of the applied ablation strategies in the recurrence and non-recurrence group can be summarized as follows: CPVI alone (31.2 vs. 37.8% in non-paroxysmal AF and 85.3 vs. 83.4% in paroxysmal AF), CPVI + LINE (44.6 vs. 42.6% in non-paroxysmal AF and 12.8 vs. 16.1% in paroxysmal AF), CPVI + CFAE (2.9 vs. 3.8% in non-paroxysmal AF and 0.3 vs. 0.3% in paroxysmal AF), and CPVI + LINE + CFAE (21.4 vs. 15.8% in non-paroxysmal AF and 1.6 vs. 0.1% in paroxysmal AF). Compared with patients without recurrence, patients with AF recurrence were more likely to suffer from ER and a longer AF duration (all *p* < 0.05). In both paroxysmal and non-paroxysmal AF, there were no significant differences in terms of age, HTN, chronic heart failure, and previous stroke/transient ischemic attack (TIA), eGFR, LAD, LVEF, types of anticoagulant, ablation strategies, and complications between recurrence and non-recurrence groups.

**TABLE 1:** Baseline characteristics.

	Non-paroxysmal AF				Paroxysmal AF			
Variable	Total (*n* = 649)	Recurrence (*n* = 276)	Non-recurrence (*n* = 373)	*P*-value	Total (*n* = 1114)	Recurrence (*n* = 367)	Non-recurrence (*n* = 747)	*P*-value
Age, years	64 (57-68)	64 (57–69)	63 (57–68)	0.385	63 (57–68)	63 (58–69)	63 (57–68)	0.487
Male, n (%)	439 (67.6)	179 (64.9)	260 (69.7)	0.192	633 (56.8)	189 (51.5)	444 (59.4)	0.012
Smoking, n (%)	133 (20.5)	58 (21.0)	75 (20.1)	0.777	203 (18.2)	73 (19.9)	130 (17.4)	0.312
Drinking, n (%)	84 (12.9)	38 (13.8)	46 (12.3)	0.590	114 (10.2)	43 (11.7)	71 (9.5)	0.252
AF duration, month	12.0 (2.4–60.0)	24.0 (4.8–60.0)	9.6 (2.4–36.0)	< 0.001	12.0 (1.2–36.0)	18.0 (2.4–48.0)	6.0 (1.2–36.0)	< 0.001
**Medical history**								
HTN, n (%)	347 (53.5)	150 (54.3)	197 (52.8)	0.699	559 (50.2)	186 (50.7)	373 (49.9)	0.814
CHF, n (%)	140 (21.6)	55(19.9)	85 (22.8)	0.381	55 (4.9)	21 (5.7)	34 (4.6)	0.397
DM, n (%)	122 (18.8)	60 (21.7)	62 (16.6)	0.099	217 (19.5)	84 (22.9)	133 (17.8)	0.044
Stroke/TIA, n (%)	99 (15.3)	43 (15.6)	56 (15.0)	0.843	120 (10.8)	37 (10.1)	83 (11.1)	0.602
CAD, n (%)	100 (15.4)	49 (17.8)	51 (13.7)	0.155	209 (18.8)	83 (22.6)	126 (16.9)	0.021
Vascular disease, n (%)	29 (4.5)	14 (5.1)	15 (4.0)	0.522	51 (4.6)	17 (4.6)	34 (4.6)	0.952
CHA2DS2-VASc score	1 (1–3)	2 (1–3)	1 (1–3)	0.128	1 (1–2)	1 (1–2)	1 (1–2)	0.129
**Procedure**								
CPVI alone, n (%)	227 (35.0)	86 (31.2)	141 (37.8)	0.079	936 (84.0)	313 (85.3)	623 (83.4)	0.419
CPVI + LINE, n (%)	282 (43.5)	123 (44.6)	159 (42.6)	0.622	168 (15.1)	47 (12.8)	121 (16.1)	0.137
CPVI + CFAE, n (%)	22 (3.4)	8 (2.9)	14 (3.8)	0.552	3 (0.3)	1 (0.3)	2 (0.3)	1.000
CPVI + LINE + CFAE, n (%)	118 (18.2)	59 (21.4)	59 (15.8)	0.069	7 (0.6)	6 (1.6)	1 (0.1)	0.01
AF termination during ablation, n (%)	184 (28.4)	77 (27.9)	107 (28.7)	0.826	1066 (95.7)	345 (94.0)	721 (96.5)	0.052
**Periprocedural complications**								
Cardiac tamponade, n (%)	3 (0.5)	0 (0)	3 (0.8)	0.364	13 (1.2)	6 (1.6)	7 (0.9)	0.470
Pseudoaneurysm, n (%)	4 (0.6)	3 (1.1)	1 (0.3)	0.418	4 (0.4)	1 (0.3)	3 (0.4)	1.0
Perioperative stroke, n (%)	4 (0.6)	3 (1.1)	1 (0.3)	0.188	1 (0.1)	1 (0.3)	0	0.329
Early recurrence, n (%)	146 (22.5)	99 (35.9)	47 (12.6)	< 0.001	150 (13.5)	101 (27.5)	49 (6.6)	< 0.001
**Medications**								
Antiplatelet, n (%)	34 (5.2)	16 (5.8)	18 (4.8)	0.583	88 (7.9)	30 (8.2)	58 (7.8)	0.812
ACEI/ARB, n (%)	251 (38.7)	118 (42.8)	133 (35.7)	0.066	354 (31.8)	120 (32.7)	234 (31.3)	0.644
B -blocker, n (%)	392 (60.4)	164 (59.4)	228 (61.1)	0.660	596 (53.5)	225 (61.3)	371 (49.74)	< 0.001
Amiodarone, n (%)	568 (87.5)	247 (89.5)	321 (86.1)	0.191	695 (62.4)	248 (67.6)	447 (59.8)	0.012
Statin, n (%)	318 (49.0)	143 (51.8)	175 (46.9)	0.218	559 (50.2)	208 (56.7)	351 (47.0)	0.002
Warfarin, n (%)	206 (31.7)	96 (34.8)	110 (29.5)	0.152	304 (27.3)	112 (30.5)	192 (25.7)	0.090
NOAC, n (%)	443 (68.3)	180 (65.2)	263 (70.5)	0.152	810 (72.7)	255 (69.5)	555 (74.3)	0.090
Diuretics, n (%)	165 (25.4)	71 (25.7)	94 (25.2)	0.880	112 (10.1)	51 (13.9)	61 (8.2)	0.003
**Echocardiogram parameters**								
LAD, mm	42 (39–45)	42 (39–45)	42 (39–45)	0.255	37 (35–40)	38 (35–40)	37 (35–39)	0.077
LVEDD, mm	48 (45–51)	48 (45–50)	48 (46–51)	0.437	47 (44–49)	46 (44–49)	47 (44–49)	0.117
LVEF,%	57 (55–59)	57 (55–59)	57 (55–58)	0.061	59 (58–59)	59 (58–59)	59 (58–59)	0.567
**Biomarkers**								
Uric Acid, μmol/L	373 (320–430)	361 (308–427)	375 (328–432)	0.081	335 (284–393)	335 (283–399)	335 (284–392)	0.814
Dyslipidemia, n (%)	350 (53.9)	157 (56.9)	193 (51.7)	0.194	642 (57.6)	222 (60.5)	420 (56.2)	0.176
eGFR, ml/(min ■1.73 m^2^)	90 (79–101)	90 (79–102)	90 (79–100)	0.612	93 (81–104)	93 (81–105)	93 (80–104)	0.974

*ACEI, angiotensin-converting enzyme inhibitors; ARB, angiotensin-converting enzyme receptor blockers; CAD, coronary artery disease; CFAE, complex fractionated electrograms; CHF, congestive heart failure; CPVI, circumferential pulmonary vein isolation; DM, diabetes mellitus; eGFR, estimated glomerular filtration rate; HTN, hypertension; LAD, left atrium diameter; LINE, linear ablation; LVEDD, left ventricular end diastolic diameter; LVEF, left ventricular ejection fraction; NOAC, new oral anticoagulants; TIA, transient ischemic attack.*

### Relationship Between Long AF Duration, Early Recurrence of Atrial Fibrillation, and Late Recurrence

**FIGURE 2:**
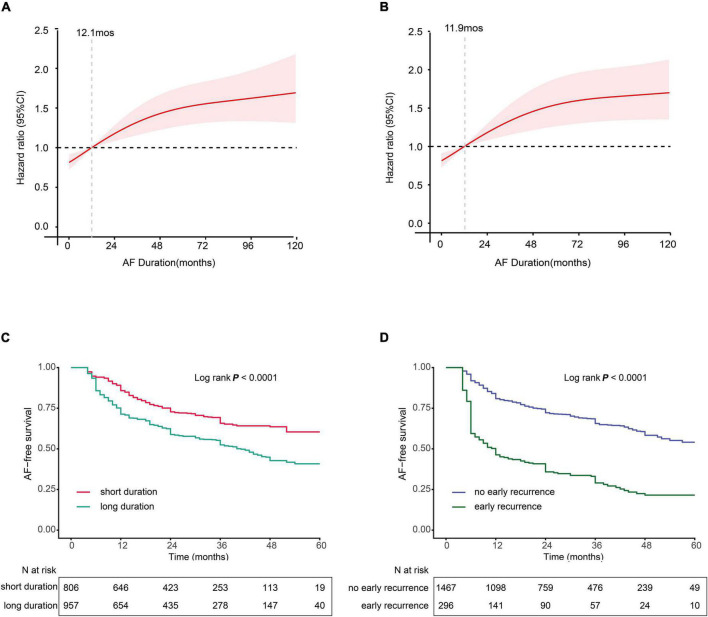
**(A,B)** Multivariable-adjusted hazard ratios (HRs) for atrial fibrillation (AF) recurrence according to levels of AF duration on a continuous scale. **(A)** Paroxysmal AF and **(B)** non-paroxysmal AF. Red lines are multivariable-adjusted HRs, with pink areas showing 95% confidence intervals (CIs) derived from RCS regressions with four knots located at the 5th, 35th, 65th, and 95th percentiles. Reference lines for no association are indicated by the dashed gray lines at an HR of 1.0. Analyses were adjusted for age, smoking, sex gender, hypertension (HTN), estimating glomerular filtration rate (eGFR), coronary artery disease (CAD), diabetes mellitus (DM), early recurrence (ER), body mass index (BMI), left atrial dimension (LAD), and left ventricular ejection fraction (LVEF). **(C,D)** Kaplan–Meier curves show the incidence of recurrence. **(C)** Kaplan–Meier survival curves for long AF duration (LAFD), the cutoff points for AF duration 12 months in both non-paroxysmal AF and paroxysmal AF. **(D)** Kaplan–Meier survival curves for ER.

We further explored the association between AF duration and late recurrence using an RCS analysis by stratifying patients into low- and high-risk groups based on the reference value of AF duration (Figures 2A,B). For both paroxysmal and non-paroxysmal AF, we observed a very narrow interval cutoff point for late recurrence. The cutoff points for late recurrence in paroxysmal AF and non-paroxysmal AF were 12.1 and 11.9 months, respectively. As a result, we defined patients with the AF course ≥ 12 months as “LAFD.”

The association between a long duration and ER of AF and the risk of recurrence is presented in Table 2. In the fully adjusted model, ER was associated with a 1.34-fold increase in AF recurrence among patients who were in the non-paroxysmal category (HR: 2.34, 95% CI: 1.82–3.01, *p* < 0.001). Similarly, we observed a 2.40-fold increase in the risk for late recurrence in paroxysmal AF (HR: 3.40, 95% CI: 2.68–4.30, *p* < 0.001). Moreover, LAFD was associated with an increase of 80.0% for the risk of late recurrence among patients with non-paroxysmal AF (HR: 1.80, 95% CI: 1.38–2.35, *p* < 0.001). In addition, we observed a 48.3% increase in the risk for late recurrence in paroxysmal AF (HR: 1.48, 95% CI: 1.20–1.84, *p* < 0.001).

**TABLE 2:** Hazard ratios (HRs) for the association between long atrial fibrillation (AF) duration and ER with AF recurrence.

	Long AF duration vs. Short AF duration			Early recurrence vs. No early recurrence		
	Model 1	Model 2	Model 3	Model 1	Model 2	Model 3
Non-paroxysmal AF	1.905 (1.470–2.469)***	1.911 (1.474–2.478)***	1.800 (1.378–2.352)***	2.500 (1.953–3.199)***	2.324 (1.812–2.981)***	2.339 (1.820–3.006)***
Paroxysmal AF	1.617 (1.309–1.997)***	1.583 (1.280–1.957)***	1.483 (1.198–1.836)***	3.480 (2.765–4.379)***	3.292 (2.613–4.148)***	3.398 (2.683–4.303)***

***p ≤ 0.001.

*Model 1, crude; Model 2, adjustment for sex and age; Model 3, full-adjustment model [adjustment for age, smoking, sex gender, hypertension (HTN), estimating glomerular filtration rate (eGFR), coronary artery disease (CAD), diabetes mellitus (DM), early recurrence (ER), body mass index (BMI), left atrial dimension (LAD), and left ventricular ejection fraction (LVEF)].*

The Kaplan–Meier survival curve analysis showed that LAFD and ER were found to significantly affect the free-AF survival rate. For instance, the incidence of AF recurrence was higher in patients with LAFD compared to those without LAFD (Figure 2C). Similarly, the incidence of late recurrence was higher in patients who experience ER compared to those without ER (Figure 2D). These findings suggest that LAFD and ER can efficiently distinguish between high- and low-risk patients.

**FIGURE 3:**
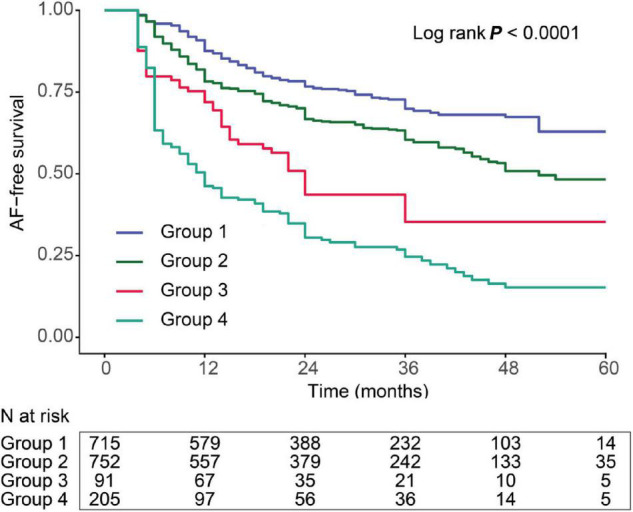
Kaplan–Meier curve showing the incidence of recurrence. All patients were divided into four categories. The lines represent the following: group 1: patients with both short AF duration and no ER (blue); group 2: patients with LAFD and no ER (green); group 3: patients with short AF duration and ER (red); and group 4: patients with both LAFD and ER (light green).

The AF recurrence rates according to the different categories of LAFD and ER statuses are shown in Figure 3. We divided patients into four groups based on the low- and high-risk subset defined by the spline curve of LAFD and ER to represent the different combinations of the two observations. These include group 1: patients with neither LAFD nor ER; group 2: patients with LAFD and no ER; group 3: patients with ER and no LAFD; and group 4: patients with both LAFD and ER. Those patients categorized in group 4 had the highest incidence of late recurrence (log-rank test, *p* < 0.001).

### The Additive Effect of Long AF Duration and Early Recurrence on the CAAP-AF Model

**FIGURE 4:**
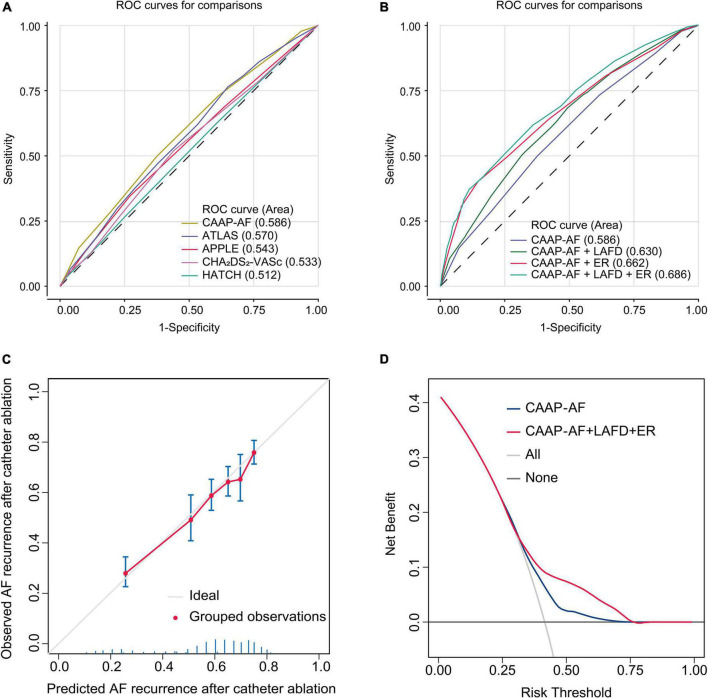
Model-comparison results of predicting the 5-year incidence of recurrence. **(A)** Receiver operating characteristics (ROC) curves of freedom from recurrence at 5 years for the different risk prediction models recommended by the guideline. **(B)** ROC curves of freedom from recurrence at 5 years for the CAAP-AF model and combined model. **(C)** Validation of the final model (CAAP-AF model + LAFD + ER) showing observed incidences of recurrence within 5 years. The diagonal gray line represents a situation of perfect prediction when the observed incidences would be identical to the predicted baseline risks. Points are drawn to represent the averages in six discretized bins, and error bars are 95% CIs for the proportion of events in each group. The rug under the plot illustrates the distribution of predictions. **(D)** Decision curve analyses (DCAs) of the CAAP-AF model and final model for 5-year recurrence incidence. The x-axis indicates the threshold incidence for recurrence at 5 years and the y-axis indicates the net benefit. The horizontal dark gray line: to assume no patients will experience the event; the light gray line: to assume all patients will experience the event. The final model had enhanced net benefit compared with the CAAP-AF model at a threshold probability of 0.4–0.75.

We performed a ROC analysis to compare the diagnostic performance of the models recommended by the guideline. The CAAP-AF score showed the highest area under the ROC curve (AUC) among the 5 scores (AUC 0.586, 95% CI: 0.559–0.613) (Figure 4A). We then tested whether combining LAFD and ER to the CAAP-AF model could improve the identification of patients with late recurrence (Figure 4B). The combination of the CAAP-AF model, LAFD, and ER (final model) had the highest AUC (0.686, 95% CI: 0.660–0.711) followed by the model of CAAP-AF and ER (0.662, 95% CI: 0.636–0.690), and CAAP-AF and LAFD (0.630, 95% CI: 0.603–0.657). To further evaluate an added prognostic discriminatory power for LAFD and ER, we assessed IDI and NRI and observed a significant incremental improvement of IDI and NRI compared to the CAAP-AF model (Table 3). Overall, the detection of late recurrence was improved by the addition of LAFD and ER to the CAAP-AF in both the NRI (NRI = 0.143, *p* < 0.001) and IDI (IDI = 0.073, *p* < 0.001) analysis.

**TABLE 3:** Comparison of different risk prediction models.

	CAAP-AF	CAAP-AF + LAFD	CAAP-AF + ER	CAAP-AF + LAFD + ER
AUC (95% CI)	0.586 (0.559–0.613)	0.630 (0.603–0.657)	0.662 (0.636–0.690)	0.686 (0.660–0.711)
*P*-value	–	<0.001	<0.001	<0.001
*C*-index (95% CI)	0.576 (0.548–0.604)	0.612 (0.586–0.637)	0.657 (0.631–0.683)	0.669 (0.645–0.693)
IDI (95% CI)	Ref	0.016 (0.005–0.032)	0.064 (0.041–0.091)	0.073 (0.049–0.101)
*P*-value	–	<0.001	<0.001	<0.001
NRI (95% CI)	Ref	0.116 (0.057–0.175)	0.201 (0.154–0.246)	0.143 (0.079–0.227)
*P*-value	–	<0.001	<0.001	<0.001

*AUC, area under ROC curve; ER, early recurrence.*

The calibration plot in Figure 4C showed that the observed frequencies and the estimated probability of late recurrence showed a good calibration curve for the risk estimation. In addition, the decision curve showed the clinical usefulness of the CAAP-AF model and final model (Figure 4D). The analysis showed that the final model had a higher net benefit at a threshold probability of 0.4–0.75.

## Discussion

In this study, we investigated the impact of LAFD and ER on the long-term prognosis of patients undergoing RFCA. We found that LAFD and ER were significantly associated with increased incidence of late recurrence after RFCA and added discriminatory capacity to the CAAP-AF model to identify patients with a high risk of late recurrence after the index RFCA.

The prevalence of recurrence for patients with AF who underwent RFCA varied from 30 to 60% (3). In our study, the late recurrence rate after RFCA was 36.5%. In this study, two variables including LAFD and ER that were not included in the CAAP-AF model were found to be independent predictors of late recurrence. Therefore, it may be reasonable to consider AF patients with LAFD and ER as candidates for a more intensive follow-up.

Atrial fibrillation often manifests as a progressive disease, and a longer AF duration is associated with heavier AF burden and atrial remodeling, both of which are known to contribute to the maintenance of AF (20, 21) and may be accompanied by serious complications and poor prognosis (22–25). A possible explanation for the relationship between AF duration and AF recurrence completes a vicious circle. The longer AF is sustained, the more it causes tissue remodeling and the greater the extent of tissue remodeling, the longer AF is sustained. This accounts for the need for appropriate patient selection and choices on anticoagulants after ablation during a follow-up.

The early treatment of atrial fibrillation for the stroke prevention (EAST-AFNET 4) trial found that early rhythm-control therapy was associated with a lower risk of adverse cardiovascular outcomes (5). However, this study did not investigate the effect of early rhythm control on later recurrence. Similarly, our study found that patients with AF of longer duration have a higher risk of recurrence, which suggested that early rhythm intervention might reduce late recurrence. Therefore, a further longitudinal study is required to confirm the impact of early rhythm control in AF recurrence following RFCA.

In the past, ER was common after the ablation procedure. Steinberg et al. reported that the incidence of ER ranges between 16 and 65%, depending on the type of ambulatory rhythm monitoring used (26). Consistent with the published data, our study showed a 16.8% incidence rate of ER. The mechanisms underlying ER are multifactorial, including post-ablation inflammation (27), short-term autonomic imbalance (28), the reconnection of PV conduction, and non-PV foci triggering (29). Increased inflammation marker is reported to be associated with ER (27, 30). This phenomenon could partly be explained by local myocardial injury as well as a systemic inflammatory process. Previous research has found that ganglionated plexus (GP) is associated with the clinical recurrence and an additional GP ablation could decrease the arrhythmia recurrence after AF ablation (28). The additional benefit may result from complete autonomic denervation by GP ablation combined with PVI. This also emphasizes the importance of autonomic imbalance on recurrence. Taken together, numerous factors affect ER through alterations in atrial myocardial conduction and refractoriness. In line with previous studies (31–34), we confirmed the experience of ER as a powerful independent predictor for late recurrence.

In most of the studies, we usually consider the first 3 months after RFCA as a blanking period, ERs during the blanking period were not appraised, because it was traditionally seen as a transient stimulatory effect of the acute inflammatory response following the histopathologic tissue damage that resulted from radiofrequency energy, not affecting long-term results. However, the 3-month definition of a blanking period has been challenged by recent studies (26, 29) with later onset of ER (the 2nd and 3rd month after RFCA) being more predictive for late recurrence. As post-ablation inflammatory phase is usually limited to a few days after RFCA (27), and the recovery of transient autonomic dysfunction is usually limited to 1 month after RFCA (35). Additionally, well-demarcated homogenous lesions could be formed within 1 week after RFCA (36, 37). Further prospective, multicenter, large sample studies on the appropriate management of ER are highly desirable, and the necessity to redefine a more clinically predictive postprocedure blanking period is emphasized.

Another interesting finding of the present study is that there is no difference between procedural strategies and late recurrence in patients with both non-paroxysmal and paroxysmal AFs, which is consistent with the Substrate and Trigger Ablation for Reduction of Atrial Fibrillation Trial Part II (STAR AF II) trial (9). This may be due to incomplete ablation or progressive atrial substrate (38). Performing additional, and perhaps unnecessary, ablation could increase the risk as well as increased exposure to fluoroscopy for the patient and operator (39). The adverse effects of x-ray radiation on human health are of increasing concern worldwide (40). The zero x-ray ablation approach is gaining increasing attention. More studies focusing on the balance between occupational radiation exposure and ablation outcome are needed in the future.

In this study, we found that LAFD and ER were independent risk factors of late recurrence, by combining them with the CAAP-AF model, we constructed a new model. Based on the analysis of our data, we endorse 12 months in paroxysmal AF and non-paroxysmal AF as the cutoff value of AF duration, and those patients with LAFD and ER should be recognized as a high-risk group for recurrence and require an intensive and strict follow-up to prevent the risk of recurrence.

Our study has some limitations. First, this was a non-randomized, observational study, and thus it is necessary to validate the results from a prospective study with larger sample size. Second, the AF duration in our study was documented based on ECG. However, asymptomatic subclinical AF is common, the ECG-based AF duration may not precisely detect AF duration. Third, the use of 24-h Holter recordings during a follow-up may overestimate the success rate. However, the method and frequency of follow-up were identical in all groups. Future research with more precise monitoring in patients with AF is needed. Also, with the ongoing technological advances in catheter ablation, treatment improvement has been achieved in recent years, there might be a difference in late recurrence across different years. However, patients were recruited continuously to reduce the bias between groups. Large well-designed RCTs are still needed to validate our findings. Finally, our study did not include all predictors of the MB-LATER score and ALARMEc score, thus we were unable to evaluate those scores.

## Conclusion

In conclusion, we found strong associations between LAFD, ER, and late recurrence in patients who underwent RFCA for AF. The combination of LAFD and ER with the CAAP-AF model significantly improved the prediction of recurrence in patients with AF who underwent RFCA, indicating that the application of these two factors to the prediction model can significantly improve discrimination for late recurrence.

## Data Availability Statement

The raw data supporting the conclusions of this article will be made available by the authors, without undue reservation.

## Ethics Statement

The studies involving human participants were reviewed and approved by the Institutional Review Board of the First Affiliated Hospital of the Dalian Medical University. Written informed consent was not required for this study, in accordance with the local legislation and institutional requirements.

## Author Contributions

XY and YX designed this study. ZL and SW were in charge of data analysis and data collection. ZL drafted the manuscript. YS and TH did the critical revision of the manuscript. LG conducted the data collection. All authors have read and approved the final manuscript.

## Conflict of Interest

The authors declare that the research was conducted in the absence of any commercial or financial relationships that could be construed as a potential conflict of interest.

## Publisher’s Note

All claims expressed in this article are solely those of the authors and do not necessarily represent those of their affiliated organizations, or those of the publisher, the editors and the reviewers. Any product that may be evaluated in this article, or claim that may be made by its manufacturer, is not guaranteed or endorsed by the publisher.
